# Feasibility and acceptability of a preoperative checklist health promotion in elective surgery in the UK: a mixed-methods study protocol

**DOI:** 10.1136/bmjopen-2025-109010

**Published:** 2025-11-13

**Authors:** Sivesh Kathir Kamarajah, Jugdeep Dhesi, Kamlesh Khunti, Krishnarajah Nirantharakumar, Clare Hughes, Joyce Yeung, Shalini Ahuja, Dion Morton, Aneel Bhangu

**Affiliations:** 1 Department of Applied Health Sciences, University of Birmingham, Birmingham, UK; 2 University of Birmingham, Birmingham, UK; 3 Ageing and Health, Guy's and St Thomas’ Hospitals NHS Trust, London, England, UK; 4 Diabetes Research Centre, University of Leicester, Leicester, England, UK; 5 Institute of Applied Health Research, King’s College London, London, England, UK; 6 University Hospitals Birmingham NHS Foundation Trust, Birmingham, England, UK; 7 Warwick Medical School, University of Warwick, Coventry, England, UK; 8 Health Services and Population Research Department, King’s College London, London, UK; 9 NIHR Global Health Research Unit on Global Surgery, Universities of Birmingham, Edinburgh and Warwick, Birmingham, UK

**Keywords:** SURGERY, Health Equity, Health Services

## Abstract

**Abstract:**

**Introduction:**

Multimorbidity or the presence of two or more long-term conditions is now common in people undergoing surgery. However, current care pathways often miss these healthcare encounters to support long-term health promotion. Therefore, there is a need for practical, scalable approaches that can be integrated into routine surgical care, for which limited solutions exist at present. We have co-designed a structured preoperative checklist to help identify and manage long-term conditions in patients listed for elective surgery. This study aims to evaluate the feasibility and acceptability of this preoperative checklist in patients undergoing elective surgery.

**Methods and analysis:**

This is a mixed-methods feasibility study in one National Health Service trust in the UK. We will recruit up to 50 adults scheduled for elective surgery and use the checklist during initial surgical clinic appointments. Quantitative data will include recruitment and retention rates, completion of the checklist and baseline clinical characteristics, analysed using descriptive statistics. Qualitative data will be collected through semistructured interviews with up to 16 patients and clinicians. These interviews will be analysed thematically, guided by the Consolidated Framework for Implementation Research. Triangulation of quantitative and qualitative data will allow us to explore fidelity, acceptability, barriers and facilitators to implementation and refine the intervention ahead of a future pilot cluster randomised trial.

**Ethics and dissemination:**

This study has received approval from the Yorkshire & The Humber - Sheffield Research Ethics Committee (approval number: 25/YH/0045). All participants will give written informed consent. Results will be published in peer-reviewed journals and shared with participants, the public and policy stakeholders.

**STRENGTHS AND LIMITATIONS OF THIS STUDY**

- The mixed-methods approach, combining quantitative and qualitative data, will provide a detailed understanding of the checklist’s practical implementation and acceptability to both patients and clinicians.
- We use prespecified progression criteria and a priori frameworks such as Reach, Adoption, Implementation and Maintenance for reporting, Carroll for fidelity and Weiners’ tool for acceptability to structure the feasibility evaluation.
- One of the limitations is this study is conducted in one National Health Service site with small sample sizes, but meaningful to gather learning ahead of wider evaluation.

## Introduction

 People are living longer, and, as a result, the number of people living with long-term health conditions such as cancer, diabetes and hypertension has increased rapidly over the past two decades.^1 2^ These conditions often occur together, known as multiple long-term conditions (MLTC) or multimorbidity. MLTC is now recognised as a major public health challenge, with 14 million adults in the UK living with MLTC.^3^,^5^ Projections suggest that the number of people affected will double or even triple in the next decade, especially among older adults.^6^ This rapid increase in the prevalence of MLTC poses challenges for health and care systems. Existing models of care, particularly in secondary care, are often designed around single diseases. This has led to fragmented and poorly coordinated care for people with MLTC, particularly during times when they are most vulnerable, such as around the time of surgery.^7^ Patients with MLTC have higher healthcare use and often experience poorer outcomes, including after surgery.^8^

Each year, 313 million people undergo surgery worldwide,^9^ and up to seven million in the UK alone.^10^ Surgery in people with MLTC is more complex, associated with higher risk and more expensive for health systems.^8^ Therefore, it is essential that the surgical journey delivers good short-term and long-term outcomes for these patients. Over the past decade, there have been advances in surgical pathways. For example, the orthogeriatric model has improved outcomes for patients undergoing surgery for hip fractures and has been adopted in many countries.^11 12^ However, similar models are infrequent in other types of surgery. Prehabilitation programmes, which prepare patients for surgery, exist, but are limited to a few centres and often focus only on the short preoperative period.^13 14^ Therefore, there is little attention paid to long-term holistic care and health promotion for people with MLTC in surgical pathways. As rates of MLTC continue to rise, especially in patients undergoing elective surgery, there remains a need for practical, scalable solutions that can be embedded into routine surgical care.^15^

To address this knowledge gap, we co-designed a preoperative checklist with key stakeholders such as patients, surgeons, anaesthetists, nurse specialists and geriatricians.^16^ This choice was informed by preparatory work: pathway mapping showed that preassessment often occurs late, leaving little time to optimise long-term conditions; a rapid scoping and a national survey suggested that screening for common conditions (eg, hypertension, diabetes) at the point of listing is uncommon; clinicians reported recognising MLTC but being unsure of the immediate next steps in busy clinics; beliefs varied about whether early optimisation was feasible and referral routes to primary care or specialist services were inconsistent.^16^ Patients and clinicians therefore prioritised a brief, structured tool to be used at the first surgical clinic after listing. The checklist brings guideline-based prompts together on one page, standardises the conversation and triggers clear actions (tests to request or retrieve, brief health-promotion advice and referrals or letters to primary care), without creating a new clinic. This checklist aims to address two linked problems: (1) surgery is a teachable moment to support long-term health for people with MLTC and (2) uncontrolled MLTC drives avoidable perioperative risk. Therefore, the ultimate impact is two-fold: (1) better long-term management and (2) fewer perioperative harms through earlier identification and action.

However, this checklist has not yet been tested in routine clinical practice. Before planning a larger pilot trial, it is important to understand if the checklist is feasible and acceptable to patients and clinicians and to identify potential barriers and facilitators to its use. Feasibility studies are an essential step in the development of complex interventions, allowing refinement of both the intervention and its implementation in real-world settings. The aim of this study is to assess the feasibility of implementing a structured preoperative checklist for health promotion and MLTC management in elective surgical patients in the National Health Service (NHS) (table 1). The primary objectives are to:

1. Determine the feasibility of the intervention, including recruitment, retention and completion rates;

**Table 1:** Mapping of objectives of study to relevant quantitative and qualitative phase and measurement throughout study

	Measure	Cohort study	Interviews (patients)	Interviews (clinicians)	Source	RE-AIM framework
Objective 1: feasibility of study						
Recruitment	Proportion of eligible patients enrolled (screened → eligible → approached → consented)	Yes	–		Screening log	Reach
Retention or attrition	Proportion of patients completing follow-ups	Yes	–		Study log	Reach
Checklist completion, adherence	Per eligible clinic visit: REDCap record or scanned paper checklist linked to ID, cross-checked versus attendance list; plus item completion %	Yes	–		REDCap; PDFs; clinic log	Reach and adoption
Objective 2: evaluate acceptability and fidelity (patients and clinicians).						
Fidelity: dose	Counts of actions: tests, advice, referrals/signposting, primary care letter; verified in notes	Yes	–	Yes	Checklist; patient records	Implementation
Fidelity: quality/ competence	Proportion of completion of checklist actions against a predefined criterion	Yes	–	–	Audit tool	Implementation
Fidelity: participant responsiveness	Patient usefulness, understanding, actions started (3–5 items)		Yes		Patient questionnaire	Implementation
Acceptability or appropriateness	*Patients: AIM means; % ≥4/5* *Clinicians: AIM means; % ≥4/5*		Yes	Yes	Patient and clinician survey	Adoption and implementation
Objective 3						
Barriers to delivery	CFIR-guided interviews; matrix with quantitative signals		–	Yes	Interview notes	Adoption &and implementation
Objective 4						
Intervention maintenance	Maintenance of health promotion		Yes - 3 months and 6 months		Maintenance	
	Improved adherence to chronic disease and medications		Yes - 3 months and 6 months		Maintenance	
	Intervention maintenance	–	–	Yes - 2 months	Maintenance	
Objective 5						
Mechanisms	New diagnosis of MLTC-research notes	Yes	Yes - 3 months and 6 months		–	
	Follow-up in primary or secondary care-research notes	Yes	Yes - 3 months and 6 months		–	
Objective 6						
Acceptability of outcome measure	Multimorbidity burden treatment questionnaire: response rate; missingness; burden comments	0 months, 3 months and 6 months			–	Implementation

MLTC, multiple long-term conditions; RE-AIM, Reach, Adoption, Implementation, Maintenance.

1. Evaluate the acceptability and fidelity of intervention delivery to patients and clinicians;
2. Understand barriers and facilitators to delivering the intervention and refine implementation strategies;
3. Assess how well the intervention can be maintained within existing surgical pathways, including links to primary care;
4. Explore how the checklist may influence current pathways and outcomes;
5. Assess the acceptability of proposed outcome measures.

This feasibility study will use mixed methods to capture both quantitative outcomes (such as recruitment and completion rates) and qualitative insights from patients and clinicians. The findings will be used to refine the checklist and its implementation and to inform the design of a future randomised trial.

## Methods and analysis

### Study design and setting

**Figure 1:**
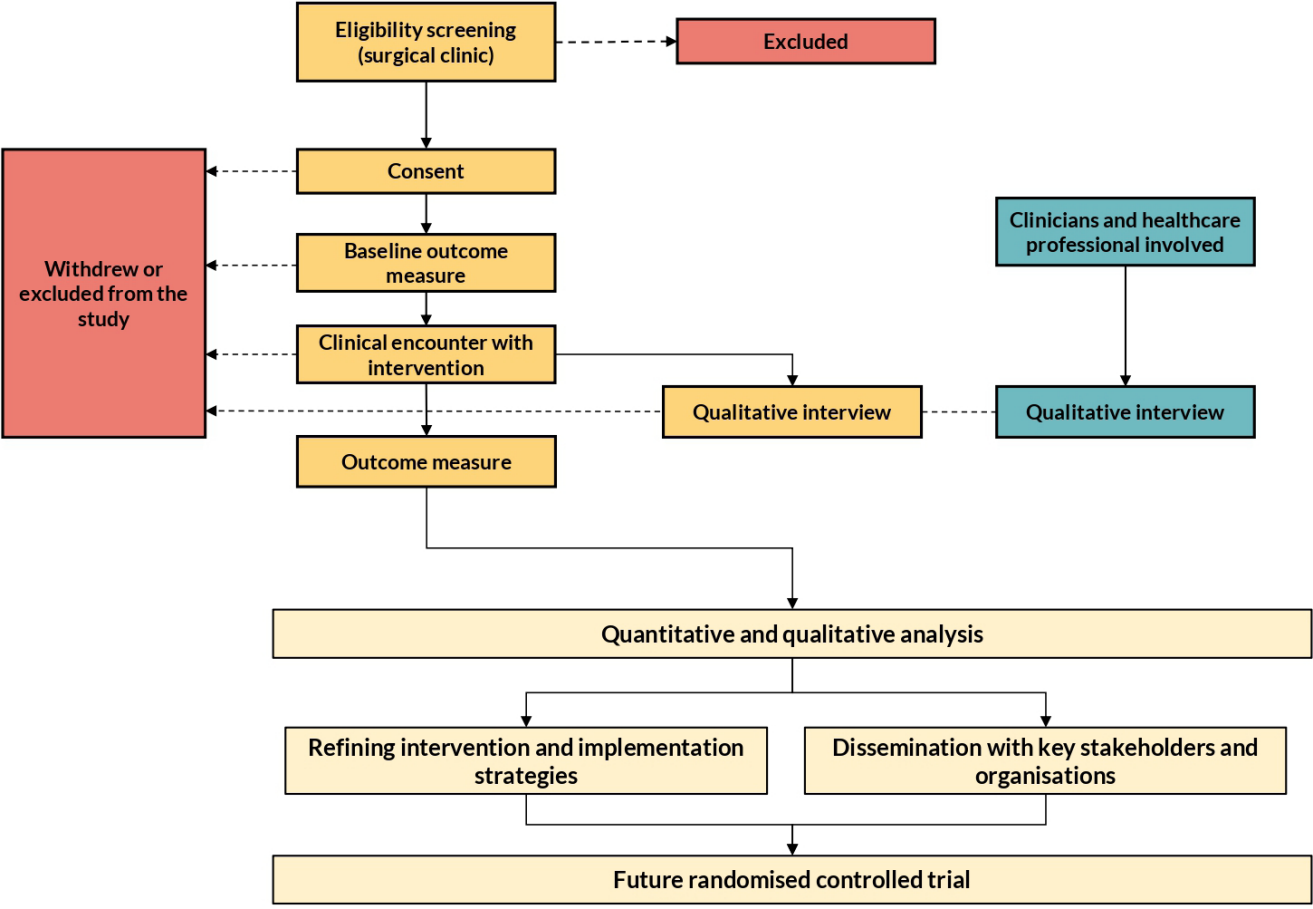
A summary flow chart of the overall plan of the mixed-methods feasibility study.

This pilot and feasibility study is a non-randomised study, and all participants will receive the intervention. The pilot and feasibility study will be consistent with guidance proposed by Eldridge *et al*^17^ and O’Cathain *et al*^18^ and reported using the adapted Standard Protocol Items: Recommendations for Interventional Trials^19^ reporting template for feasibility studies (online supplemental file 1). A mixed methods approach will be undertaken to assess the feasibility of the preoperative checklist, drawing on principles from the National Institute of Health Research and Medical Research Council Complex intervention framework (figure 1).^20^ The study will take place in one large NHS hospital site, University Hospital Birmingham NHS Trust. The preoperative checklist will be implemented in surgical clinics at hospitals within the trust, and follow-up data will be collected as part of routine care. The planned study period is from September 2025 to December 2025. Recruitment will take place over 3 months, with follow-up continuing for up to 6 months post enrolment. Qualitative interviews will be conducted during and after this period, with data analysis taking place alongside data collection and following study completion. A detailed schedule of procedures is presented in table 2.

**Table 2:** Summary schedule of procedures for the mixed-methods feasibility study

	Time point			
	t_0_ (Surgical clinic)	t_1_ (1–2 months)	t_1_ (3 months)	t_2_ (6 months)
Enrolment				
Eligibility screen	x			
Patient consent (overall study)	x			
Patient consent (interview)	x			
Intervention				
Clinical use of checklist	x			
Assessments				
Baseline	x			
Follow-up and PROMs	x		x	x
Interview (clinician)		x		
Interview (patient)			x	

PROM, Patient Reported Outcome Measure.

### Patient and public involvement

Patients were involved in commenting on and reviewing the initial application to the University of Birmingham. Together with patients and public group and expert clinician group, who were involved at the outset of the NIHR funding application, they have iteratively designed this feasibility study. For this part of the study, patients have been involved in designing the topic guide and the patient lead will be involved to review the coding framework from the interviews. They will also work with them to produce a lay summary of the results for dissemination to study participants.

### Intervention

The intervention consists of a structured preoperative checklist, which has been co-designed with patients, surgeons, anaesthetists, nurse specialists and geriatricians.^16^ The checklist is a brief, clinician-delivered tool used during the surgical clinic that integrates five components: (1) early identification prompts (diagnosed conditions, current treatments, recent Blood pressure (BP), Haemoglobin 1AC (HbA1c) where relevant, smoking, alcohol, physical activity, weight/body mass index, frailty indicators) to surface long-term health needs at the first post-listing contact; (2) action prompts aligned to guidance (eg, request or retrieve recent tests; confirm accurate BP; consider HbA1c if diabetes is suspected; reconcile medicines; flag polypharmacy concerns) so that the discussion leads to specific next steps; (3) brief health promotion advice on smoking and weight management to structure a short, patient-centred conversation and agree one achievable action; (4) coordination triggers to standardise onward care (tick-box referrals/signposting to primary care or specialist services and an auto-templated letter to primary care summarising agreed actions, tests and responsibilities) and (5) follow-up flag to revisit the checklist within 6 months where a review is planned, recording actions completed and any barriers. Key materials include a one-page paper (tick-boxes plus brief free text for the plan) and a local referral directory (primary care advice and guidance, specialist clinics, community services). The checklist promotes health-related discussion by scripting key topics and supports coordination by generating a short, standardised plan and primary care letter during the same visit, without creating a new clinic or lengthening the pathway. The template checklist is presented in online supplemental file 2.

The checklist is intended for all adult patients (≥18 years) listed for elective upper or lower gastrointestinal surgery who attend participating surgical clinics during the study period. Exclusions are day-case minor procedures that do not involve a preoperative assessment, non-elective/emergency presentations and patients unable to consent in English without an interpreter when none is available (sites may include translated materials where feasible). Clinicians are prompted to use the checklist at the first clinic visit after listing (‘post-listing clinic’) via a sticker on the printed clinic list, an EHR banner or smart-phrase where available and the REDCap start page. Use is strongly encouraged as part of routine documentation; non-use and the reason (eg, urgent add-on, interpreter delay, clinic over-run) are recorded. Where a review is planned, the checklist may be revisited at a subsequent follow-up clinic within 6 months to document progress on agreed actions. The checklist is delivered by the surgical team, usually a consultant surgeon or clinical nurse specialist, after a 60 min orientation covering purpose, components, worked cases and how to record and communicate the plan; a local clinical champion is available for on-the-shoulder support during the first fortnight.

### Participants

The target population consists of adults aged 18 years or older who are listed for elective surgery for benign or malignant conditions at participating NHS sites. Eligible participants must be able to give informed consent and participate in study procedures, including completion of the checklist and, if invited, an interview. Patients scheduled for emergency surgery and those unable to give consent will be excluded from the study. For the qualitative interviews, participants must be able to take part in an interview in English, with translation services available if required. In addition to patients, the study includes the clinicians who deliver the intervention in routine clinics: consultant surgeons and clinical nurse specialists working in participating upper and lower gastrointestinal services. Clinician users receive a 60 min orientation before study launch covering the purpose of the checklist, its five components, clinic workflow, documentation (paper/REDCap) and communication of the agreed plan to primary care. Training includes two worked cases and a quick-reference guide; a local clinical ‘champion’ provides on-the-shoulder support during the first fortnight. During the study period, checklist use is strongly encouraged at the first clinic visit after listing for all eligible adult patients. Sites implement simple prompts (eg, clinic list sticker, EHR smart-phrase/flag, REDCap start page). Use is not mandated; when it is not used, the reason (eg, urgent add-on, interpreter delay, clinic over-run) is recorded to inform feasibility and implementation planning. Where a review is planned, the checklist may be revisited at a subsequent clinic within 6 months to document progress.

### Recruitment and consent process

Patients will be recruited via the NHS hospitals providing surgical specialty services across the UK. Patients attending upper or lower gastrointestinal surgical clinics will be screened for potential inclusion. Patients will be approached by a member of the direct care team, either a clinical nurse specialist or by a member of the surgical team. Recruitment will take place at a routine clinical visit. Patients will be provided with a copy of the study patient information sheet which will give full details of the study. Interested patients will be asked for consent for the clinical team to enter their contact details onto the secure REDCap data capture system^21^ hosted by the University of Birmingham. This initial collection of data is through implied consent. They will also be asked for consent for participating in an interview of up to 1 hour. They will then be contacted to answer any questions that they may have and to arrange a suitable time for the interview. Each participant will be allocated a unique identification number for the purposes of the study for the purposes of interview transcription. Participating sites will also be asked to display a poster advertising the study in appropriate waiting rooms. For the interviews with healthcare professionals, they will be recruited directly by the research team. They will also be asked for consent to participate in an interview of up to 1 hour and arrange a suitable time for the interview. Each participant will be allocated a unique identification number for the purposes of the study for the purposes of interview transcription. Participating sites will also be asked to display a poster advertising the study in appropriate waiting rooms. Prior to recruitment, patients will be provided with a patient information sheet and will be asked to complete a consent form for both the feasibility study and interview. The process for obtaining informed consent will be in accordance with Good Clinical Practice. The consent form must be completed before the scheduled time of the interview. Both the patient and clinician consent form is presented in onlinesupplemental files 3  4, respectively.

### Outcomes

Since this is a feasibility study, the primary outcome is the measuring of the overall feasibility of the intervention. To keep the study pragmatic and low-burden, we prioritised a small set of core feasibility signals such as adoption, fidelity (adherence, dose, quality or competence, participant responsiveness) and acceptability. Other useful constructs (eg, compatibility with workflow, clinician self-efficacy, adaptability) will be explored qualitatively using the interview framework rather than as additional questionnaires, to reduce duplication and respondent fatigue in routine clinics. This approach is consistent with early-stage feasibility work and our prespecified progression criteria; domains that surface as important will be carried forward for targeted quantitative assessment in the next phase. Therefore, we align outcomes and measures to the six study objectives. The overall reporting is organised with RE-AIM (Reach, Adoption, Implementation, Maintenance). Briefly, fidelity is defined using Carroll’s framework (adherence, dose, quality/competence, participant responsiveness), acceptability / appropriateness / feasibility are measured with Weiner’s Acceptability Intervention Measure (AIM) and explored qualitatively with Theoretical Framework of Acceptability (TFA)-guided interviews and Consolidated Framework for Implementation Research (CFIR) is used for barriers and facilitators. Table 1 maps each objective to its outcomes, measures, sources, timing and RE-AIM domains. Onlinesupplemental files 5^8^ provide (1) the 10% audit criteria, (2) patient questionnaire items (including AIM/IAM) and (3) interview guides.

### Sample size

Since this is a feasibility study, no formal size is needed. We will recruit up to 50 patients on the waiting list for planned gastrointestinal surgery from the surgical or preoperative clinics for the feasibility study. We will recruit for a maximum of 3 months. This will allow for the project’s objectives to be achieved and is consistent with other UK feasibility trials.^22^ The qualitative interviews will be completed with clinicians delivering the intervention and the participants receiving it. We aim to recruit 16 participants (patients and clinicians) for the interviews. There is no minimum number of patients for the cohort study. The final sample size for the interview will be dependent on iterative analysis to achieve ‘saturation’. Saturation is the point when no new themes or ideas are identified from interviews. The numbers needed to reach saturation can be <10 or >20. Based on previous works, we estimate that this will be sufficient to reach data saturation and meet the pragmatic objectives of the study.^23^ If saturation is not reached at this point, then further interviews will be undertaken.

### Data collection and management

Quantitative data will be collected from all enrolled participants at baseline and at follow-up clinic appointments up to 6 months after recruitment. Baseline data will include demographic characteristics (age, gender, ethnicity, smoking status, past medical history) and MLTC burden as measured by the Multimorbidity Burden Treatment Questionnaire.^24 25^ At each clinic visit, information regarding new diagnoses of long-term conditions and follow-up care (in primary or secondary settings) will be recorded. Checklist completion will also be monitored as part of routine clinical documentation. Data on recruitment, retention and attrition will be collected prospectively. Qualitative data will be obtained through semistructured interviews with a purposive sample of up to 16 participants, including both patients and healthcare professionals involved in the intervention. Interviews will explore participants’ experiences with the checklist, perceived value, barriers and facilitators to its use and recommendations for improvement. All interviews will be audiorecorded, transcribed verbatim and anonymised for analysis. Interview topic guides have been developed with input from patient and public representatives and presented in onlinesupplemental files 7  8. All study data will be stored securely on the University of Birmingham REDCap servers. Access will be restricted to authorised study team members only. Participant data will be de-identified using unique study identifiers, and personal contact information will be deleted once the study is complete. Audio recordings and transcripts will be handled in accordance with university data protection policies and will be deleted at the end of the study. There are no plans to share data with third parties or for use in other research.

### Statistical analysis

Quantitative data will be summarised using descriptive statistics, including means and SD or medians and ranges for continuous variables, and frequencies and proportions for categorical variables. No formal hypothesis testing will be performed as this is a feasibility study. Qualitative data from semistructured interviews will be developed and analysed using two complementary frameworks using thematic analysis, following the approach described by Braun and Clarke.^26^ Coding and theme development will be guided by the CFIR framework^27^ to systematically identify barriers and facilitators to implementation and TFA and map questions to its seven domains (affective attitude, burden, ethicality, intervention coherence, opportunity cost, perceived effectiveness, self-efficacy). NVivo software will be used to manage and code qualitative data. Integration of findings from both quantitative and qualitative components will be undertaken using a triangulation approach. This will provide a comprehensive understanding of the feasibility, fidelity and acceptability of the checklist, as well as areas for refinement prior to a future definitive trial.

## Discussion

The rising prevalence of multimorbidity presents an urgent challenge for health systems worldwide, especially in the context of elective surgery. While there have been some advances, most surgical pathways continue to focus on single diseases, leading to fragmented and poorly coordinated care for people with multiple health problems. Opportunities for health promotion and management of long-term conditions are often missed, and care for surgical patients with multimorbidity tends to be reactive rather than proactive. This study aims to address this gap by evaluating a structured preoperative checklist, co-designed with patients, clinicians and stakeholders. The checklist is intended as a practical, scalable tool to support the identification and management of common long-term conditions such as diabetes and hypertension in patients listed for elective surgery, embedding health promotion earlier in the surgical pathway. By intervening before the preoperative clinic, there may be greater opportunity to address modifiable risk factors and support better patient outcomes.

The proposed study is timely, and its feasibility design is well-suited to capture the complexities of introducing a new intervention in real-world NHS settings. Using a mixed-methods approach that combines quantitative data with in-depth qualitative interviews, the study will provide a comprehensive understanding of the checklist’s acceptability, fidelity and integration into busy clinical workflows. The use of the CFIR framework will help to systematically identify barriers and facilitators to implementation, towards a future cluster randomised trial. Further findings from the study will help to iteratively refine the intervention according to the ADAPT guidance,^28^ aligned to the National Institute of Health Research and Medical Research Council Complex Intervention framework^20^ and maximise generalisability to incoming sites for a future pilot trial. A key strength of this study is its strong focus on patient and public involvement throughout the development of the intervention and the research process. Involving patients, clinicians and other stakeholders from the outset has helped ensure that the checklist is relevant, acceptable and grounded in real clinical needs. Conducting the study at two NHS hospital sites will enhance the generalisability of findings, and purposive sampling for interviews will allow for a broad range of views to be explored.

However, there are some important limitations. As a feasibility study, the sample size is small, and the work is not powered to assess clinical effectiveness or patient outcomes. However, this study will provide baseline data and learning to support wider scale evaluation. The study is limited to one NHS hospital site and may not fully represent all clinical settings. The realities of NHS surgical clinics, such as time pressures and competing demands, may also affect the fidelity and sustainability of checklist use. Despite purposive sampling, there is a risk that some patient or clinician perspectives will be under-represented. Finally, we did not quantify some feasibility constructs (eg, detailed compatibility, self-efficacy, adaptability) beyond targeted interview prompts; this was a pragmatic choice to minimise burden in busy clinics and will be addressed in the next evaluation phase if indicated. Despite these limitations, the study will generate critical learning about the practicalities and challenges of implementing a new tool for multimorbidity management in surgery. If the intervention proves feasible and acceptable, this work will provide a strong foundation for a future definitive trial to assess effectiveness and impact on patient outcomes. More broadly, the findings may inform policy and clinical practice in the NHS and beyond, contributing to the ongoing shift towards patient-centred, integrated and preventative care for people with MLTC undergoing surgery.

## Supplementary material

10.1136/bmjopen-2025-109010 online supplemental file 1

10.1136/bmjopen-2025-109010 online supplemental file 2

10.1136/bmjopen-2025-109010 online supplemental file 3

10.1136/bmjopen-2025-109010 online supplemental file 4

10.1136/bmjopen-2025-109010 online supplemental file 5

10.1136/bmjopen-2025-109010 online supplemental file 6

10.1136/bmjopen-2025-109010 online supplemental file 7

10.1136/bmjopen-2025-109010 online supplemental file 8
