# Supplementary material for: Feasibility and acceptability of a preoperative checklist health promotion in elective surgery in the UK: a mixed-methods study protocol
Source: BMJ Open. 2025 Nov 13;15(11):e109010. doi: 10.1136/bmjopen-2025-109010 (PMC12625896; doi:10.1136/bmjopen-2025-109010)
Supplement: online supplemental file 2 [file bmjopen-15-11-s002.pdf]

PIONEER: Multimorbidity surgical passport for long-term health

[HOSPITAL LOGO]

Patient name:

Patient ID:

Date of birth:

Planned procedure? **Yes** ☐

Surgery name:

Section 1: If current smoker?

Provide brief advice to patients ☐

Advise patient to visit local pharmacy for cessation service ☐

Section 2: If BMI >30kg/m^2

Provide brief advice to patients ☐

Refer to dietician on PICS via 'Referral tab' for walk-in service ☐

Section 3: Diabetes assessment

Step 1 If unknown diabetes or HbA1c >3 months, request HbA1c ☐

Step 2 ***If not known diabetes*** & HbA1c >48mmol/mol, refer to GP for review ☐

If known diabetes with HbA1c 58-69mmol/mol, inform GP for review ☐

If known diabetes with HbA1c >69mmol/mol, refer to diabetes nurse specialist via PICS ☐

Section 4: Hypertension assessment

If blood pressure ≥140/90mmHg, inform GP for review ☐

Section 5: If age >65 and one of the following: clinical frailty score (CFS; see overleaf)* is ≥5, known dementia, or prior history of delirium

Refer to POPS team via PICS HCOP Referral and request out-patient frailty pre- operative review ☐

*CFS is only validated in patients aged 65 and above.
