# Supplementary material for: Feasibility and acceptability of a preoperative checklist health promotion in elective surgery in the UK: a mixed-methods study protocol
Source: BMJ Open. 2025 Nov 13;15(11):e109010. doi: 10.1136/bmjopen-2025-109010 (PMC12625896; doi:10.1136/bmjopen-2025-109010)
Supplement: online supplemental file 4 [file bmjopen-15-11-s004.docx]

**Participant Study ID code:**

**Consent Form**

## **Study Title: Evaluating Feasibility of a Preoperative Checklist for Opportunistic Long-term Health Promotion in Patients Undergoing Elective Surgical Care in the NHS**

We are asking if you would like to take part in a research study to establish what the acceptability of the checklist intervention and potential barriers to implementing this. Before you consent to participating in the study we ask that you read the participant information sheet and mark each box below with your initials if you agree.

| To be completed online by the participant and electronically signed | |
| --- | --- |
|  | Please initial each box to show agreement |
| 1. I confirm that I have read the information sheet and fully understand what is expected of me within this study |  |
| 1. I confirm that I have had the opportunity to ask any questions and to have them answered. |  |
| 1. I understand that my participation is voluntary and that I am free to withdraw at any time without giving any reason. |  |
| 1. *I understand that my interview will be audio recorded as part of this research and agree to my interview being audio recorded* |  |
| 1. *I understand that the recordings of my interview will be shared with a contracted transcription service, and agree for my data to be shared for this purpose.* |  |
| 1. *I understand that relevant sections of my medical notes and data collected during the study, may be looked at by individuals from the University of Birmingham, from regulatory authorities or from the NHS Trust, where it is relevant to my taking part in this research. I give permission for these individuals to have access to my records* |  |
| 1. I understand that the interview will be audio recorded and once my interview has been transcribed and included in the study database, my responses cannot be removed from reports |  |
| 1. I understand that my contact details are required to participate in the study, and this will only be used by the research team to conduct tasks related to the study including organising the interview. |  |
| 1. I understand that none of my personal identifiable data will be shared in the reports of this study. |  |
| 1. I understand that anonymised quotes may be used in reports or publications. |  |
| 1. I agree to the study team retaining the transcript of my interview for a period of up to 10 years |  |
| 1. I would like to receive a copy of the final results of the study, and agree for the research team to keep my contact details to be able to send this to me   Preferred method of contact: |  |
| 1. I agree to take part in this study. |  |

___________________ ______________________ ________________

Participant’s Name Signature Date

(*print name*)

___________________ ______________________ ________________

Researcher’s Name Signature Date

(*must be on the*

*delegation log*)

*Original for the study file, one copy for the participant*
