# Supplementary material for: Feasibility and acceptability of a preoperative checklist health promotion in elective surgery in the UK: a mixed-methods study protocol
Source: BMJ Open. 2025 Nov 13;15(11):e109010. doi: 10.1136/bmjopen-2025-109010 (PMC12625896; doi:10.1136/bmjopen-2025-109010)
Supplement: online supplemental file 5 [file bmjopen-15-11-s005.docx]

**Fidelity notes review**

**A. Adherence (already captured quantitatively; confirm in audit)**

- A1. Checklist present and linked to the specific clinic visit (Yes/No).
- A2. Required fields completed ≥80% (Yes/No).

**B. Dose / Exposure** *(evidence that the checklist triggered action)*

- B1. **Tests** requested or retrieved when indicated (e.g., no recent BP/HbA1c): Evidence present (Yes/No).
- B2. **Brief advice** recorded for at least one behaviour domain when flagged (smoking/alcohol/activity/weight): Evidence present (Yes/No).
- B3. **Referral/signposting** made when indicated (e.g., uncontrolled BP; suspected diabetes; polypharmacy concern): Evidence present (Yes/No).
- B4. **GP letter** summarising plan generated/filed: Evidence present (Yes/No).

**C. Quality / Competence** *(alignment of actions with prompts)*
Score **Pass** if **all** applicable criteria met; otherwise **Fail**.

- C1. Elevated BP recorded (or undocumented/no recent reading); plan documented (repeat reading, home BP, GP follow-up, or clinic re-check).
- C2. Diabetes suspected (symptoms, risk factors) or known with no recent HbA1c; HbA1c ordered/retrieved or GP contact documented.
- C3. Polypharmacy/high-risk medicines (e.g., anticoagulants, steroids, hypoglycaemics); medicines reconciliation or plan documented.
- C4. Tobacco/alcohol above brief-intervention thresholds; brief advice documented **and** signposting/leaflet or referral if available.
- C5. Physical inactivity or obesity flagged; brief advice documented **and** one agreed action (e.g., walking plan, local service).

**D. Participant responsiveness** *(patient-centred evidence in record)*

- D1. Plain-language explanation of the plan recorded (Yes/No).
- D2. Agreed action captured in patient words or using “teach-back” phrasing (Yes/No).

**Overall quality score:** Pass if **C** passes and at least **two** of **B1-B4** are Yes. Record reasons for fail (free text).
