# Supplementary material for: Feasibility and acceptability of a preoperative checklist health promotion in elective surgery in the UK: a mixed-methods study protocol
Source: BMJ Open. 2025 Nov 13;15(11):e109010. doi: 10.1136/bmjopen-2025-109010 (PMC12625896; doi:10.1136/bmjopen-2025-109010)
Supplement: online supplemental file 6 [file bmjopen-15-11-s006.docx]

**Patient acceptability questionnaire**

**Instructions:** “These questions are about the plan you agreed in surgical clinic. Please think about that clinic visit.”

**1. Participant-responsiveness items (fidelity)**

1. **Usefulness:** “The clinic conversation helped me understand what I can do for my health before and after surgery.”
2. **Understanding:** “I understood the plan we agreed in clinic.”
3. **Action started:** “I have started at least one action from the plan (for example a test, an exercise, or contacting my GP).”
4. **Support:** “I know who to contact if I have questions about the plan.”
   **Response scale for items 1-4:** 1 Strongly disagree, 2 Disagree, 3 Neither, 4 Agree, 5 Strongly agree.
5. **Barriers (tick any):** couldn’t get appointment / cost of travel / work or caring duties / felt unsure what to do / side-effects or symptoms / other (free text).
6. **Free text (optional):** “What helped or made it harder to follow the plan?”

**2. Weiner implementation outcome measures (patient AIM/IAM)**

Use the 4-item versions, scored 1–5 (Strongly disagree to Strongly agree). Compute mean score; report %≥4.

**AIM (Acceptability):**

- “I like the checklist-guided clinic conversation.”
- “The checklist is appealing to me.”
- “The checklist seems fitting for people like me.”
- “I welcome this way of planning care.”

**IAM (Appropriateness):**

- “The checklist fits my needs before surgery.”
- “The checklist seems suitable for people with long-term conditions.”
- “The checklist seems applicable in my situation.”
- “The checklist seems like a good match for surgical clinic.”

**Optional short demographics:** age band; sex; number of long-term conditions (self-rated: 0–1 / 2–3 / 4+).

**Scoring:** Produce means (SD), medians (IQR), and % of respondents scoring ≥4 (“agree/strongly agree”) for each scale; missing data reported.

**Clinician acceptability survey**

**3.1 Weiner measures**

- **AIM, IAM, FIM** (4 items each; 1-5). Use the standard validated wording (clinic-facing).

**FIM (Feasibility) example items:**

- “The checklist seems implementable in our clinic.”
- “The checklist seems possible to use.”
- “The checklist seems doable within our workflow.”
- “The checklist seems easy to use.”

**3.2 Additional feasibility constructs**

- **Compatibility:** “The checklist fits with our clinic workflow.” (1–5)
- **Self-efficacy:** “I am confident using the checklist as intended.” (1–5)
- **Adaptability:** “It is easy to tailor the checklist to our subspecialty.” (1–5)
- **Time burden:** REDCap auto-timer (mins) or categorical backup: <3 / 3–5 / >5.
- **Impact on flow:** “Using the checklist disrupted my clinic.” (reverse-coded, 1–5)
- **Open text:** “One change that would make the checklist easier to use…”

**Scoring:** Means (SD), %≥4, and distribution of time.
