# Supplementary material for: Feasibility and acceptability of a preoperative checklist health promotion in elective surgery in the UK: a mixed-methods study protocol
Source: BMJ Open. 2025 Nov 13;15(11):e109010. doi: 10.1136/bmjopen-2025-109010 (PMC12625896; doi:10.1136/bmjopen-2025-109010)
Supplement: online supplemental file 7 [file bmjopen-15-11-s007.docx]

**Title:** Evaluating feasibility of a preoperative checklist for opportunistic long-term health promotion in people with undergoing elective surgical care in the NHS

Interview Topic guide - Patients

**[Before turning on the recorder]**

- Introduce myself
- Ask the participant how they would like to be called (first or last name)
- Go through the participant information sheet and the consent form and confirm participants are happy to continue participation
- Remind participants that they are happy to pause the interview at any time as well as withdraw from the study at any time
- Remind participants that the interview will be audio recorded and transcribed by a TP professional transcription company (third party), which holds a confidentiality agreement with University of Birmingham. This means your data will be safe.
- Explain that for confidentiality purposes, participants are encouraged to not reveal any personally identifiable information (such as name, date of birth, address) during the interview. If such information happens to be mentioned during the interview this will be removed accordingly during the transcription process, replacing the instances with tags such as [Name], [Place]. TP Transcription Limited will not use any of these personal data as they hold a confidentiality contract University of Birmingham, meaning your data will be protected.
- In the event the patient becomes upset or needs a break, the interview will be paused and can be resumed when they are ready or stopped if they do not wish to continue.
- Explain that the researcher is going to note down things that they want to come back to
- Reiterate there’s no right or wrong answer etc.
- The interview will take about an hour. Questions before we start?

**[Turn on the recorder]**

**Introduction**

1. Can you tell me a bit about yourself?

Prompts:

- Ethnicity
- Occupation
- Current health

**Understanding the Whole Journey**

1. Could you walk me through your journey from first being told you might need surgery up to now?
   - How did you first learn you might need surgery?
   - How did you get referred?
   - What appointments or steps did you have before the surgical clinic?
   - What information or support did you receive?
   - Were there points where you felt lost, worried, or unsupported?

**Navigation and Coordination**

1. How easy was it to know what you needed to do next?
   - Who helped you understand your pathway?
   - Was it clear who to contact if you had questions?
   - Did you feel your GP and hospital team were working together?

**Experience Managing Long-term Conditions**

1. Can you comment on the care you receive for your long-term condition(s) from your GP or at hospital?
   - Any challenges managing your health as you prepared for surgery?
   - Any problems with medication, appointments, or advice?
   - What or who helped you most to manage these challenges?

**Points of Transition**

1. How was the transition from your GP to the hospital surgical team?
   - Did your GP provide any summary or handover to the surgical team?
   - Was anything missed or repeated?

**What Matters Most to You**

1. What is most important to you about how care is provided, especially while you are waiting for surgery?
   - Is there anything you would like to change or improve?
   - If so, how?

**Gaps and Suggestions for Improvement**

1. Was there any help or information you wish you’d had, but didn’t?
   - What would have made things easier?
   - Any delays or duplication you noticed?
2. What single change would have made the biggest difference to your experience?

**Acceptability and Use of the Checklist**

1. Are you aware of the preoperative checklist system?
   1. If yes, what do you think of it?
   2. Did anyone explain it to you? Who?
   3. Did you find it appropriate and suitable for the surgical clinic?
   4. Would you like to see it used more, or in other settings?
   5. Was there anything you were not happy about?
2. If the checklist was used, did it help you talk more easily with your doctors or nurses about your health conditions?
   - Would it help to have a similar checklist at other points, like at your GP surgery, or after surgery?
   - Who do you think should complete this checklist with patients?

**Impact of the Checklist/System Change**

1. Has the checklist (or any other part of the pathway) had any effect on your health or treatment, either in hospital or primary care?
   - Did it change anything in how your care was organised or decisions made?
2. Would you like to see the checklist system continue?
3. If it wasn’t continued, what would be the most important parts to keep, or add, to the current system?

***Closing***

- *Look at notes- are there any questions to revisit?*

So, we are coming to the end of the interview now and I have asked you everything that I wanted to. Is there anything else you would like to tell me about?

How do you feel about being involved with this research? What do you think about the questions asked? Is there anything else you would like to add or think I should have asked? Is there anything you would like to ask me?

Thank you very much for taking the time to give us your views. Your contribution to this research will help us to think about how best to support patients with multimorbidity through the surgical pathways in the NHS.

***Turn off recorder:***

-  *Give thanks*

-  *Reiterate what happens next*
