# Supplementary material for: Feasibility and acceptability of a preoperative checklist health promotion in elective surgery in the UK: a mixed-methods study protocol
Source: BMJ Open. 2025 Nov 13;15(11):e109010. doi: 10.1136/bmjopen-2025-109010 (PMC12625896; doi:10.1136/bmjopen-2025-109010)
Supplement: online supplemental file 8 [file bmjopen-15-11-s008.docx]

**Title:** Evaluating feasibility of a preoperative checklist for opportunistic long-term health promotion in people with undergoing elective surgical care in the NHS

Interview Topic guide - HCPs

**[Before turning on the recorder]**

- Introduce myself
- Ask the HCP how they would like to be called (first or last name)
- Go through the participant information sheet and the consent form and confirm participants are happy to continue participation
- Remind participants that they are happy to pause the interview at any time as well as withdraw from the study at any time
- Remind participants that the interview will be audio recorded and transcribed by a TP professional transcription company (third party), which holds a confidentiality agreement with University of Birmingham. This means your data will be safe.
- Explain that for confidentiality purposes, participants are encouraged to not reveal any personally identifiable information (such as name, date of birth, address) during the interview. If such information happens to be mentioned during the interview this will be removed accordingly during the transcription process, replacing the instances with tags such as [Name], [Place]. TP Transcription Limited will not use any of these personal data as they hold a confidentiality contract University of Birmingham, meaning your data will be protected.
- Do you have any questions before we begin?
- The purpose of the interview is to understand if the intervention is acceptable and if so, maximise the opportunity to implement the intervention in a future trial.
- Can you confirm that you are happy for the interview to be recorded?
- Please can you sign to consent to us recording the interview?

**[Turn on the recorder]**

**Background & Experience *(All)***

- Can you tell me a bit about your experience working with patients with multiple long-term conditions?
  - Profession, years in practice, main settings
  - Main challenges (patient, system, personal)
    - *[CFIR: Outer Setting—Patient Needs; Inner Setting—Culture; Characteristics of Individuals]*

**Current Perceptions of Care & Priorities for Improvement**

*> All HCPs:*

- How would you describe the current quality of care for patients with multiple long-term conditions in the surgical pathway?
  - What works well?
  - Biggest challenges or gaps?
  - Most important priorities in caring for these patients?
  - Are there places where patients “fall through the cracks”?
    - *[CFIR: Inner Setting—Implementation Climate; Outer Setting—Patient Needs]*
- If you could change or improve anything about current care, what would it be?
  - “Quick wins” or system-level changes needed?
    - *[CFIR: Process—Reflecting & Evaluating; Inner Setting—Culture]*

*> Primary Care / GP only:*

- How do you see your role in preparing patients with long-term conditions for surgery?
  - *[CFIR: Characteristics of Individuals—Role, Identity]*
- What works well, and what could be improved, in the way primary and secondary care communicate about elective surgery?
  - *[CFIR: Process—Networks & Communication]*
- How easy is it for you to know where your patient is on their surgical journey?
  - *[CFIR: Outer Setting—Patient Needs; Process—Networks & Communication]*
- Are you usually informed when a patient you look after is being considered for surgery or attending a surgical clinic?
  - *[CFIR: Process—Networks & Communication]*
- How do you coordinate medication or care changes with the surgical team?
  - *[CFIR: Inner Setting—Implementation Climate; Process—Engaging]*
- Is there anything that would make it easier for you to support your patients before and after surgery?
  - *[CFIR: Inner Setting—Readiness for Implementation]*

**Experience with the Checklist**

**> All HCPs:**

- **What is your experience of using the preoperative checklist?**
  - **Did it fit with your workflow?**
    - ***[CFIR: Intervention—Complexity, Compatibility]***
  - **Did you like it/find it helpful?**
    - ***[CFIR: Intervention—Relative Advantage]***
  - **Did you adapt it?**
    - ***[CFIR: Intervention—Adaptability]***
  - **Would you change content, timing, or delivery?**
    - ***[CFIR: Intervention—Design Quality, Adaptability]***
  - **Anything missing, or issues with suitability?**
    - ***[CFIR: Intervention—Design Quality; Outer Setting—Patient Needs]***

**B. Surgeons/Nurse Specialists only:**

- **Did the checklist affect your clinical decisions or discussions with patients/families?**
  - ***[CFIR: Process—Engaging; Intervention—Relative Advantage]***
- **Was it practical in your clinic (time, flow, space)?**
  - ***[CFIR: Inner Setting—Implementation Climate; Intervention—Complexity]***

**C. Primary Care / GP only:**

- **Were you ever informed about checklist results or recommendations for your patients?**
  - ***[CFIR: Process—Networks & Communication; Outer Setting—Patient Needs]***
- **Did any information from the checklist change what you did or prompt you to follow up with a patient?**
  - ***[CFIR: Intervention—Relative Advantage; Process—Engaging]***
- **Would you like to see something like this used or shared in primary care?**
  - ***[CFIR: Intervention—Adaptability; Outer Setting—Patient Needs]***
- **What format of information transfer (letter, electronic, phone) would work best for you?**
  - ***[CFIR: Process—Networks & Communication; Inner Setting—Implementation Climate]***

**4. Barriers & Solutions *(All)***

- **What difficulties or barriers did you face in delivering or using the checklist?**
  - **IT, time, staffing, duplication?**
    - ***[CFIR: Inner Setting—Available Resources, Implementation Climate]***
  - **Patient engagement?**
    - ***[CFIR: Outer Setting—Patient Needs]***
  - **Leadership or organisational support?**
    - ***[CFIR: Inner Setting—Leadership Engagement, Culture]***
- **Any workarounds or adaptations that helped?**
  - ***[CFIR: Process—Adaptation, Engaging]***
- **What made it easier to use/embed? Any positive drivers?**
  - ***[CFIR: Process—Champions, Engaging]***
- **Were there times it was not possible or relevant to use?**
  - ***[CFIR: Process—Executing, Adaptability]***

**5. Maintenance & Improvement *(All)***

- **What would encourage you to keep using the checklist?**
  - ***[CFIR: Process—Reflecting & Evaluating; Inner Setting—Implementation Climate]***
- **What would you continue, change, or do differently?**
  - ***[CFIR: Process—Reflecting & Evaluating; Intervention—Adaptability]***
- **How did local circumstances (staffing, clinic flow, organisational issues) affect use?**
  - ***[CFIR: Inner Setting—Readiness for Implementation, Structural Characteristics]***
- **Any differences between clinics/settings?**
  - ***[CFIR: Outer Setting—External Policy & Incentives; Process—Networks & Communication]***

**6. Spread & Generalisability *(All)***

- **Would the checklist work in other patient groups, specialties, or settings (e.g. primary care, after surgery)?**
  - ***[CFIR: Intervention—Adaptability; Outer Setting—Patient Needs; Process—Planning]***
- **Who should be responsible for completing the checklist?**
  - ***[CFIR: Process—Roles, Champions; Inner Setting—Networks & Communication]***

**7. Closing *(All)***

- **Anything else you’d like to share about your experience?**
  - ***[CFIR: Process—Reflecting & Evaluating]***
- **How did you find being involved in this research?**
- **Anything we’ve missed, or you’d like to ask me?**

***Closing***

- *Look at notes- are there any questions to revisit?*

So, we are coming to the end of the interview now and I have asked you everything that I wanted to. Is there anything else you would like to tell me about?

How do you feel about being involved with this research? What do you think about the questions asked? Is there anything else you would like to add or think I should have asked? Is there anything you would like to ask me?

Thank you very much for taking the time to give us your views. Your contribution to this research will help us to think about how best to support patients with multimorbidity.

***Turn off recorder:***

-  *Give thanks*

-  *Reiterate what happens next*
