# Supplementary material for: Feasibility and acceptability of a preoperative checklist health promotion in elective surgery in the UK: a mixed-methods study protocol
Source: BMJ Open. 2025 Nov 13;15(11):e109010. doi: 10.1136/bmjopen-2025-109010 (PMC12625896; doi:10.1136/bmjopen-2025-109010)
Supplement: Supplementary file 9 [file bmjopen-2025-109010.reviewer_comments.pdf]

PEER REVIEW HISTORY

BMJ Open publishes all reviews undertaken for accepted manuscripts. Reviewers are asked to complete a checklist review form and are provided with free text boxes to elaborate on their assessment. These free text comments are reproduced below.

ARTICLE DETAILS

Title (Provisional)

Feasibility and acceptability of a preoperative checklist health promotion in elective surgery in the United Kingdom: A mixed-methods study protocol

Authors

Kamarajah, Sivesh; Kathir Kamarajah, Sivesh; Hughes, Clare; Dhesi, Jugdeep; Khunti, Kamlesh; Nirantharakumar, Krishnarajah; Yeung, Joyce; Ahuja, Shalini; Morton, Dion; Bhangu, Aneel

---

VERSION 1 - REVIEW

**Reviewer**	**1**
**Name**	**Ruzycki, Shannon**
**Affiliation**	**University of Calgary, Department of Medicine**
**Date**	**05-Sep-2025**
**COI**	**None**

Background:

I wasn't sure which problem the authors are using their checklist to solve (using surgery as an opportunity to address medical comorbidities OR addressing medical comorbidities so that they don't contribute to worse outcomes after surgery OR both). I suggest more clearly defining this underlying issue for readers, as this would be the ultimate outcome of the checklist.

The authors should outline why they selected a preoperative checklist as an intervention to address the problem described in the first two paragraphs of the Background section. What type of background investigation into the problem was done? Did they have evidence (formal research or anecdotally from stakeholders) that preoperative healthcare providers were not properly screening for MLTC? Were patients not being recognized as having multiple comorbidities? Were patients being recognized as having MLTC but healthcare teams did not know how to do health promotion in the preoperative period? What if the true underlying problem is that surgeons and nurses can recognize MLTC but there are no clinics or interventions that can address these risks in the preoperative period? Or the

underlying problem could be that surgeons and nurses don't believe that preoperative medical optimization is possible or necessary in their setting?

Methods:

I would appreciate more information about the intervention - how does it "promote health-related discussions and support coordination of care early in the surgical pathway"? Is it simply a checklist of comorbidities or are there other tools incorporated?

Is the intervention meant to be used for every patient or are there eligibility criteria? How does a healthcare worker know if the checklist is meant to be used?

Moving the Intervention subsection to before the Participants section would be helpful.

The Participants subsection, more information is needed about who the healthcare providers are, given that the Intervention is delivered by the healthcare worker. Do they receive special training on how to use the checklist? Is the checklist mandatory in their clinics/setting, or do they decide whether or not to use it?

The Outcomes section requires more information. How will checklist completion be determined? Is this a physical copy of each checklist (per patient?) that the team will be checking or is it self-reported be the healthcare workers? How will fidelity be assessed from the perspective of patients?

The authors should describe the conceptual framework that they are using to assess fidelity (e.g., Carroll 2007 or Breitenstein 2012) - for example, there is a difference between completing the checklist (adherence) and completing the checklist properly (competence), in particular if the checklist is complex (e.g., would a nurse clinician or surgeon use the checklist correctly?).

Similarly, the framework proposed by the authors using to guide interviews is not meant to understand acceptability (CFIR is for barriers and facilitators for implementation, which is also important but does not specifically assess the domains of acceptability). I suggest the authors explore work by Weiner et al (Implementation Science 2017) on implementation outcome measures. A study protocol should outline how the interview questions will be determined (or provide an interview guide if possible) and how the interviews will be analyzed. Implementation science has many theories and frameworks to guide intervention evaluation and one should be used to guide interview development and analysis.

The authors should describe why feasibility is being assessed with fidelity and appropriateness only - what about other considerations like compatibility, self-efficacy, adaptability, etc? I suggest also using balancing measures like cost (e.g., time taken in the appointment to use the intervention rather than just monetary cost) as feasibility outcomes. Additional potential outcomes that should be considered are described in work by Teresi et al (Med Care 2022).

VERSION 1 - AUTHOR RESPONSE

Reviewer 1

1. Background
    a. I wasn't sure which problem the authors are using their checklist to solve (using surgery as an opportunity to address medical comorbidities OR addressing medical comorbidities so that they don't contribute to worse outcomes after surgery OR both). I suggest more clearly defining this underlying issue for readers, as this would be the ultimate outcome of the checklist.

Response: Thank you for raising this important question. We now state plainly that the checklist addresses two linked problems: (1) surgery is a teachable moment to support long-term health for people with MLTC; and (2) uncontrolled MLTC drives avoidable perioperative risk. The "ultimate outcome" is therefore twofold: (i) better long-term management; and (ii) fewer perioperative harms through earlier identification and action. To improve clarity of the paper, we have added the following into the **introduction [lines 114-118]**:

"This checklist aims to address two linked problems: (i) surgery is a teachable moment to support long-term health for people with MLTC; and (ii) uncontrolled MLTC drives avoidable perioperative risk. Therefore, the ultimate impact is two-fold: (i) better long-term management; and (ii) fewer perioperative harms through earlier identification and action."

    b. The authors should outline why they selected a preoperative checklist as an intervention to address the problem described in the first two paragraphs of the Background section. What type of background investigation into the problem was done? Did they have evidence (formal research or anecdotally from stakeholders) that preoperative healthcare providers were not properly screening for MLTC? Were patients not being recognized as having multiple comorbidities? Were patients being recognized as having MLTC but healthcare teams did not know how to do health promotion in the preoperative period? What if the true underlying problem is that surgeons and nurses can recognize MLTC but there are no clinics or interventions that can address these risks in the preoperative period? Or the underlying problem could be that surgeons and nurses don't believe that preoperative medical optimization is possible or necessary in their setting?

Response: We now summarise the background work that led us to a checklist: pathway mapping, a rapid scoping, a national survey, and stakeholder co-design. These showed (i)

late pre-assessment and little time to optimise, (ii) low screening at listing, (iii) uncertainty about "what to do next" in clinic, (iv) variable beliefs about whether early optimisation is possible, and (v) inconsistent referral routes. Patients and clinicians jointly prioritised a short preoperative checklist as a pragmatic solution that scripts the conversation and triggers clear next actions without lengthening appointments. We also acknowledge that clinicians often recognise MLTC but lack mechanisms (tools, time, referral options) to act, precisely what the checklist addresses. We have added the following into the **methods** **[lines 104-118]:**

"This choice was informed by preparatory work: pathway mapping showed that pre-assessment often occurs late, leaving little time to optimise long-term conditions; a rapid scoping and a national survey suggested that screening for common conditions (e.g., hypertension, diabetes) at the point of listing is uncommon; clinicians reported recognising MLTC but being unsure of the immediate next steps in busy clinics; beliefs varied about whether early optimisation was feasible; and referral routes to primary care or specialist services were inconsistent.[16] Patients and clinicians therefore prioritised a brief, structured tool to be used at the first surgical clinic after listing. The checklist brings guideline-based prompts together on one page, standardises the conversation, and triggers clear actions (tests to request or retrieve, brief health-promotion advice, and referrals or letters to primary care), without creating a new clinic. This checklist aims to address two linked problems: (i) surgery is a teachable moment to support long-term health for people with MLTC; and (ii) uncontrolled MLTC drives avoidable perioperative risk. Therefore, the ultimate impact is two-fold: (i) better long-term management; and (ii) fewer perioperative harms through earlier identification and action."

2. Methods
    a. I would appreciate more information about the intervention - how does it "promote health-related discussions and support coordination of care early in the surgical pathway"? Is it simply a checklist of comorbidities or are there other tools incorporated?

Response: We now specify that the intervention is more than a checklist. It is a brief, scripted tool with five integrated components: (1) early identification prompts; (2) action prompts aligned to guidance; (3) brief health-promotion advice for smoking and weight management; (4) coordination triggers (standard referral/signposting plus an auto-templated GP letter summarising actions); and (5) a follow-up flag to revisit agreed actions. We also list the materials (1-page REDCap form, quick-reference guide, referral directory) and explain how these components enable health conversations and cross-sector coordination without creating a new clinic.

We have added the following to the methods **[lines 172-190]**:

“The checklist is a brief, clinician-delivered tool used during the surgical clinic that integrates five components: (i) early identification prompts (diagnosed conditions, current treatments, recent BP/HbA1c where relevant, smoking, alcohol, physical activity, weight/BMI, frailty indicators) to surface long-term health needs at the first post-listing contact; (ii) action prompts aligned to guidance (e.g., request or retrieve recent tests; confirm accurate BP; consider HbA1c if diabetes is suspected; reconcile medicines; flag polypharmacy concerns) so that the discussion leads to specific next steps; (iii) brief health promotion advice on smoking and weight management to structure a short, patient-centred conversation and agree one achievable action; (iv) coordination triggers to standardise onward care (tick-box referrals/signposting to primary care or specialist services and an auto-templated letter to the GP summarising agreed actions, tests, and responsibilities); and (v) Follow-up flag to revisit the checklist within six months where a review is planned, recording actions completed and any barriers. Key materials include a one-page paper (tick-boxes plus brief free text for the plan) and a local referral directory (GP advice & guidance, specialist clinics, community services). The checklist promotes health-related discussion by scripting key topics and supports coordination by generating a short, standardised plan and GP letter during the same visit, without creating a new clinic or lengthening the pathway. The template checklist is presented in Supplementary file S2.”

b. Is the intervention meant to be used for every patient or are there eligibility criteria? How does a healthcare worker know if the checklist is meant to be used?

Response: We now state that use is a universal offer for eligible adults listed for elective GI surgery in participating clinics. We specify simple inclusions/exclusions, when to use it (first post-listing clinic), how staff are prompted (clinic list stickers/EHR banner/REDCap start page), and that reasons for non-use are logged to inform feasibility.

We have added the following into the methods **[lines 192-206]**:

“The checklist is intended for all adult patients (≥18 years) listed for elective upper or lower gastrointestinal surgery who attend participating surgical clinics during the study period. Exclusions are day-case minor procedures that do not involve a preoperative assessment, non-elective/emergency presentations, and patients unable to consent in English without an interpreter when none is available (sites may include translated materials where feasible). Clinicians are prompted to use the checklist at the first clinic visit after listing (“post-listing clinic”) via a sticker on the printed clinic list, an EHR banner or smart-phrase where

available, and the REDCap start page. Use is strongly encouraged as part of routine documentation; non-use and the reason (e.g., urgent add-on, interpreter delay, clinic over-run) are recorded. Where a review is planned, the checklist may be revisited at a subsequent follow-up clinic within six months to document progress on agreed actions. The checklist is delivered by the surgical team, usually a consultant surgeon or clinical nurse specialist, after a 60-minute orientation covering purpose, components, worked cases, and how to record and communicate the plan; a local clinical champion is available for on-the-shoulder support during the first fortnight."

c. Moving the Intervention subsection to before the Participants section would be helpful.

Response: Thank you, this has been updated.

d. The Participants subsection, more information is needed about who the healthcare providers are, given that the Intervention is delivered by the healthcare worker. Do they receive special training on how to use the checklist? Is the checklist mandatory in their clinics/setting, or do they decide whether or not to use it?

Response: We have expanded Participants to specify clinician users (consultant surgeons and clinical nurse specialists), their brief training, and how use is expected during the study. Use is strongly encouraged as part of routine documentation at the first post-listing clinic, but not mandated; reasons for non-use are logged to inform feasibility.

We have added the following into the methods **[lines 215-227]**:

"In addition to patients, the study includes the clinicians who deliver the intervention in routine clinics: consultant surgeons and clinical nurse specialists working in participating upper and lower gastrointestinal services. Clinician users receive a 60-minute orientation before study launch covering the purpose of the checklist, its five components, clinic workflow, documentation (paper/REDCap), and communication of the agreed plan to primary care. Training includes two worked cases and a quick-reference guide; a local clinical 'champion' provides on-the-shoulder support during the first fortnight. During the study period, checklist use is strongly encouraged at the first clinic visit after listing for all eligible adult patients. Sites implement simple prompts (e.g., clinic list sticker, EHR smart-phrase/flag, REDCap start page). Use is not mandated; when it is not used, the reason (e.g., urgent add-on, interpreter delay, clinic over-run) is recorded to inform feasibility and implementation planning. Where a review is planned, the checklist may be revisited at a subsequent clinic within six months to document progress."

e. The Outcomes section requires more information. How will checklist completion be determined? Is this a physical copy of each checklist (per patient?) that the team will be checking or is it self-reported be the healthcare workers? How will fidelity be assessed from the perspective of patients?
f. The authors should describe the conceptual framework that they are using to assess fidelity (e.g., Carroll 2007 or Breitenstein 2012) - for example, there is a difference between completing the checklist (adherence) and completing the checklist properly (competence), in particular if the checklist is complex (e.g., would a nurse clinician or surgeon use the checklist correctly?).
g. Similarly, the framework proposed by the authors using to guide interviews is not meant to understand acceptability (CFIR is for barriers and facilitators for implementation, which is also important but does not specifically assess the domains of acceptability). I suggest the authors explore work by Weiner et al (Implementation Science 2017) on implementation outcome measures. A study protocol should outline how the interview questions will be determined (or provide an interview guide if possible) and how the interviews will be analyzed.

Response: Thank you for these comments and you are correct. Given the use of different measures and frameworks, we have aligned outcomes and measures directly to our stated objectives. Broadly, we have kept RE-AIM as the organising taxonomy, where each signal of feasibility sits, which is the primary aim of the study. Fidelity is assessed using Carroll's framework (adherence, dose, quality/competence, participant responsiveness). Acceptability are measured with Weiner's brief tools and explored qualitatively using the Theoretical Framework of Acceptability. CFIR is used only to analyse barriers and facilitators. Checklist completion is defined per eligible clinic visit from REDCap or a scanned paper form cross-checked against the clinic attendance list (not clinician self-report). Patient-reported fidelity (usefulness, understanding, actions started) has been added.

To this extent, we have updated the following methods **[lines 255-271]**
"Since this is a feasibility study, the primary outcome is the measuring the overall feasibility of the intervention. To keep the study pragmatic and low-burden, we prioritised a small set of core feasibility signals such as adoption, fidelity (adherence, dose, quality or competence, participant responsiveness), and acceptability. Other useful constructs (e.g., compatibility with workflow, clinician self-efficacy, adaptability) will be explored qualitatively using the interview framework rather than as additional questionnaires, to reduce duplication and respondent fatigue in routine clinics. This approach is consistent with early-stage feasibility

work and our pre-specified progression criteria; domains that surface as important will be carried forward for targeted quantitative assessment in the next phase. Therefore, we align outcomes and measures to the six study objectives. The overall reporting is organised with RE-AIM (Reach, Adoption, Implementation, Maintenance). Briefly, fidelity is defined using Carroll's framework (adherence, dose, quality/competence, participant responsiveness), acceptability / appropriateness / feasibility are measured with Weiner's AIM/IAM/FIM and explored qualitatively with TFA-guided interviews; and CFIR is used for barriers and facilitators. **Table 1** maps each objective to its outcomes, measures, sources, timing, and RE-AIM domains. **Supplementary Files S5-8** provides (i) the 10% audit criteria, (ii) patient questionnaire items (including AIM/IAM), and (iii) interview guides."

h. Implementation science has many theories and frameworks to guide intervention evaluation and one should be used to guide interview development and analysis.

Response: We have specified that interview development and analysis are guided by two complementary frameworks: TFA for acceptability and CFIR for barriers/facilitators. We use these frameworks to write the topic guides, build the codebook, and structure analysis. This keeps the approach parsimonious and aligned to our objectives. We have added the following to the methods **[lines 312-325]**:

"Quantitative data will be summarised using descriptive statistics, including means and standard deviations or medians and ranges for continuous variables, and frequencies and proportions for categorical variables. No formal hypothesis testing will be performed as this is a feasibility study. Qualitative data from semi-structured interviews will be developed and analysed using two complementary frameworks using thematic analysis, following the approach described by Braun and Clarke.[24] Coding and theme development will be guided by the CFIR framework[25] to systematically identify barriers and facilitators to implementation and Theoretical Framework of Acceptability (TFA) and map questions to its seven domains (affective attitude, burden, ethicality, intervention coherence, opportunity cost, perceived effectiveness, self-efficacy). NVivo software will be used to manage and code qualitative data. Integration of findings from both quantitative and qualitative components will be undertaken using a triangulation approach. This will provide a comprehensive understanding of the feasibility, fidelity, and acceptability."

i. The authors should describe why feasibility is being assessed with fidelity and appropriateness only - what about other considerations like compatibility, self-efficacy, adaptability, etc? I suggest also using balancing measures like cost

(e.g., time taken in the appointment to use the intervention rather than just monetary cost) as feasibility outcomes. Additional potential outcomes that should be considered are described in work by Teresi et al (Med Care 2022).

Response: Thank you for the suggestion. We agree these constructs (compatibility, self-efficacy, adaptability, costs) are relevant to feasibility. However, in this study protocol, we have limited quantitative measurement to a small set of core signals such as adoption, fidelity (Carroll), and acceptability to minimise respondent load, avoid duplication across frameworks, and preserve clinic flow in a live service setting. Issues such as compatibility, self-efficacy, and adaptability will be examined qualitatively using TFA/CFIR-guided interviews, which is appropriate at this stage and aligns with our pre-specified progression criteria. If these domains emerge as material barriers, they will be prioritised for quantitative testing in the subsequent pilot or definitive evaluation. We have clarified this rationale in methods and acknowledged the trade-off in limitations.

(i) **Methods [lines 255-263]**

"Since this is a feasibility study, the primary outcome is the measuring the overall feasibility of the intervention. To keep the study pragmatic and low-burden, we prioritised a small set of core feasibility signals such as adoption, fidelity (adherence, dose, quality or competence, participant responsiveness), and acceptability. Other useful constructs (e.g., compatibility with workflow, clinician self-efficacy, adaptability) will be explored qualitatively using the interview framework rather than as additional questionnaires, to reduce duplication and respondent fatigue in routine clinics. This approach is consistent with early-stage feasibility work and our pre-specified progression criteria; domains that surface as important will be carried forward for targeted quantitative assessment in the next phase."

(ii) **Limitations [lines 365-368]**

"Finally, we did not quantify some feasibility constructs (e.g., detailed compatibility, self-efficacy, adaptability) beyond targeted interview prompts; this was a pragmatic choice to minimise burden in busy clinics and will be addressed in the next evaluation phase if indicated."
